# RNA-sequencing predicts a role of androgen receptor and aldehyde dehydrogenase 1A1 in osteosarcoma lung metastases

**DOI:** 10.1038/s41388-024-02957-x

**Published:** 2024-02-15

**Authors:** Tanya E. Heim, Margaret L. Hankins, Rebekah Belayneh, Nerone Douglas, Vu Dinh, Murali Kovvur, David N. Boone, Vrutika Ukani, Sumail Bhogal, Vaidehi Patel, Taylor M. A. Moniz, Kelly M. Bailey, Ivy John, Karen Schoedel, Kurt R. Weiss, Rebecca J. Watters

**Affiliations:** 1 University of Pittsburgh Department of Orthopaedic Surgery, Pittsburgh, PA USA; 2 University of Pittsburgh Department of Biomedical Informatics, Pittsburgh, PA USA; 3 Columbia University with Trinity College, Dublin, UK; 4 UPMC Hillman Cancer Center Academy, Pittsburgh, PA USA; 5 University of Pittsburgh School of Medicine, Department of Pediatrics, Pittsburgh, PA USA; 6 University of Pittsburgh Department of Pathology, Pittsburgh, PA USA

**Keywords:** Bone cancer, Sarcoma, Gene expression

## Abstract

One-third of pediatric patients with osteosarcoma (OS) develop lung metastases (LM), which is the primary predictor of mortality. While current treatments of patients with localized bone disease have been successful in producing 5-year survival rates of 65–70%, patients with LM experience poor survival rates of only 19–30%. Unacceptably, this situation that has remained unchanged for 30 years. Thus, there is an urgent need to elucidate the mechanisms of metastatic spread in OS and to identify targetable molecular pathways that enable more effective treatments for patients with LM. We aimed to identify OS-specific gene alterations using RNA-sequencing of extremity and LM human tissues. Samples of extremity and LM tumors, including 4 matched sets, were obtained from patients with OS. Our data demonstrate aberrant regulation of the androgen receptor (AR) pathway in LM and predicts aldehyde dehydrogenase 1A1 (ALDH1A1) as a downstream target. Identification of AR pathway upregulation in human LM tissue samples may provide a target for novel therapeutics for patients with LM resistant to conventional chemotherapy.

## Introduction

Osteosarcoma (OS) is the most common primary bone malignancy of adolescents and children. While current treatments for patients with localized bone disease are associated with 5-year survival rates of 65–70%, patients who present with or develop lung metastases (LM) experience poor survival rates of only 19–30% [1]. One-third of pediatric patients with OS develop LM, which is the primary predictor of mortality [2]. The current approach to treat both localized and metastatic OS involves surgical excision of the primary lesion with a combination of neoadjuvant and adjuvant chemotherapy to target known or suspected metastatic disease. This standard of care has been employed for over 30 years, and despite numerous attempts to develop more effective therapies, the prognosis for patients with LM remains unchanged and unacceptably low [1]. There is a paucity of understanding of the molecular mechanism of metastasis in OS. This unmet need presents the opportunity to identify novel, targetable pathways which could enable the development of more precise treatments for patients with OS LM. We aim to identify gene alterations and pathways in OS that may function as drivers of LM.

Notch signaling is a key regulator of cell proliferation, differentiation, and fate. Therefore, this signaling pathway has been a promising area of cancer research for many years [3]. The development and progression of several types of cancers, including breast, prostate, and sarcoma, have been linked to the dysregulation of Notch signaling [3–5]. Additionally, overexpression of Notch signaling proteins may be related to increased aldehyde dehydrogenase (ALDH) activity and metastatic behavior in OS cells [6]. The superfamily of ALDH enzymes catabolizes exogenous and endogenous aldehydes to minimize cellular oxidative stress [7]. Previous studies from our group have demonstrated elevated ALDH activity and expression in OS cells exhibiting higher proliferation, migration, and invasion in vitro [6, 8, 9].

Androgen receptor (AR) is a member of the nuclear receptor family, functioning as a ligand-regulated transcription factor [10]. Its expression has been noted in both normal and malignant tissues, including soft tissue sarcoma and OS, and has been hypothesized to be involved in the progression of OS [11]. Clinically, OS occurs more frequently in males than females (1.5:1). This increased prevalence of OS among males has been consistently reported for several different races as well [12]. Furthermore, many studies found male OS patients to have worse 5-year [12–16] or event-free [17] survival compared to females. In fact, males with OS have a higher incidence of distant disease and metastases compared to female patients [13] and may be less sensitive to chemotherapy as seen in similar cancers [18]. OS also tends to affect patients during periods of rapid growth. These demographics have led to the postulation that sex hormones may play a role in OS pathogenesis [18, 19]. RNA-sequencing (RNA-seq) studies that compare OS primary and recurrent tumors of the extremity to LM have been limited, mainly because patient samples, particularly metastatic tissues, are difficult to obtain.

Next-generation sequencing and genome-wide association studies have yielded considerable information about genes and pathways that promote both the pathogenesis and metastatic potential of OS [2, 20]. Previous findings have demonstrated that dysregulation of p53 and Rb play a key role in OS proliferation [21, 22]. In addition, Pan Liu et al. identified estrogen receptor 1 (ESR1), notch homolog protein 3 (NOTCH3), and caspase 1 (CASP1) as important OS-associated genes [23]. However, in addition to these individual gene markers, considerable aneuploidy and chromosomal structural disruption also appear to play a role in OS’s unique pathology [24]. More than half of OS tumors display structural genetic rearrangements, including amplifications, deletions, and translocations [25, 26]. Chromosomal gains at 6p, 8q, and 17p have been identified [27, 28], as have epigenetic changes involving alterations in DNA copy number, methylation, and mRNA expression [10]. Genomic instability is a well-known hallmark of cancer and structural variation has been exploited therapeutically in multiple cancer types [29]. However, despite the recent discoveries about the genomic character of OS, the genotypic relationship between primary, recurrent, and LM tumors remains to be elucidated.

We hypothesized that LM from patients with OS exhibit unique genetic alterations that are not present in primary or recurrent tumors and that these genetic changes drive the metastatic process and can thus be targeted therapeutically. Using RNA-seq on archived patient tumor samples, we aimed to identify changes in gene expression that enable primary bone tumor cells to spread and establish LM. To validate our genomic findings, we tested the efficacy of the AR inhibitor, enzalutamide, to reduce OS migration in vitro. Our study evaluates the genomic and molecular importance of AR in OS LM.

## Results

### ecRNA-seq of extremity osteosarcomas and LMs reveals DEG subsets

**Fig. 1:**
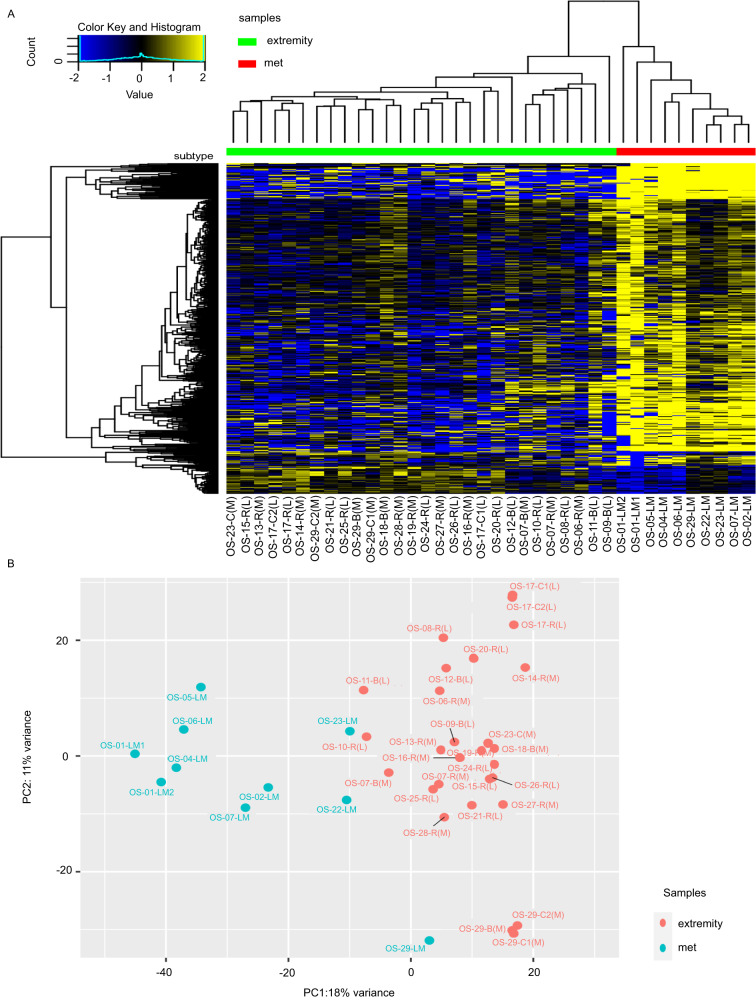
Differentially expressed genes in all extremity and lung metastases. **A** The heatmap (yellow, high relative expression; blue, low relative expression) of log2normCPM values demonstrates that LM, below the red bar, exhibit defined clusters of upregulated genes. 673 genes were differentially expressed (FDR-adjusted *P* < 0.01), fold-change = 1.5, and TPM expression profiles in the tenth percentile. **B** Principal component analysis also demonstrated distinct clustering of LM samples (blue) and extremity tumor samples (red). Analyses performed with unpaired samples, which included all extremity tumors vs. all lung metastases (*n* = 38 samples).

**Fig. 2:**
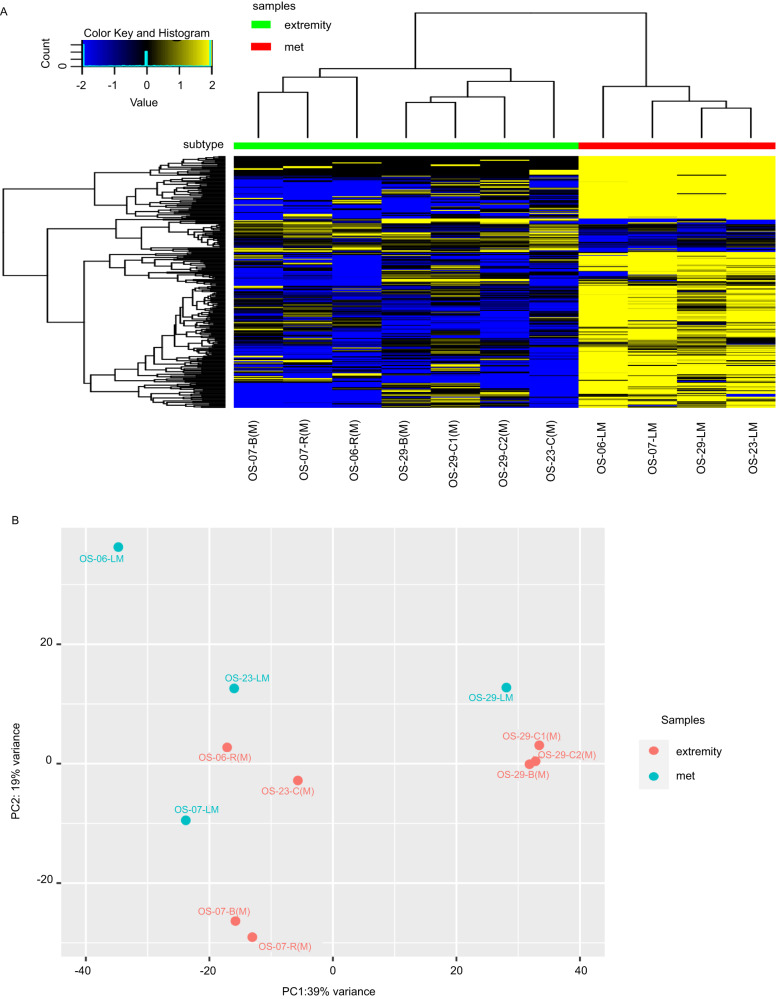
Differentially expressed genes in matched extremity tumors and lung metastases. **A** The heatmap demonstrates that LM, below the red bar, exhibit defined clusters of upregulated genes. 316 genes were differentially expressed (FDR-adjusted *P* < 0.01), fold-change = 1.5, and TPM expression profiles in the tenth percentile. **B** Principal component analyses also demonstrated distinct clustering of LM samples (blue) and extremity tumor samples (red). Analyses performed with paired samples, which included only patient-matched extremity tumors vs. lung metastases (*n* = 4 sample sets).

We performed exome capture RNA-sequencing (ecRNA-seq) to evaluate the differential gene expression between the primary and recurrent OS tumors and LM. Two analyses were performed to identify DEGs between extremity OS and LM tumors using DESeq2 (v1.24.0): (1.) All extremity tumors versus all LMs (unpaired analysis) (Fig. 1A, B), and (2.) All patient-matched extremity tumors versus LMs (paired analysis) (Fig. 2A, B). The heatmaps demonstrate a significant number of genes with different expression in LM compared to extremity tumors (yellow, high relative expression; blue, low relative expression). Median-centered, log2normCPM values demonstrate that LM, below the red bar, exhibit defined clusters of upregulated genes. The unpaired and paired analyses demonstrated 673 and 316 DEGs, respectively (FDR-adjusted *P* < 0.01, fold-change = 1.5, and TPM expression of 1).

Principal component analysis (PCA) plots for analysis of all genes show that the primary and recurrent tumors of different patients were more like each other than they were to their respective patient-matched LM. However, Fig. 1B shows primary and recurrent tumors for OS-29 clustered with each other as well as with their respective LM. The other paired samples (OS-06, OS-07, and OS-23) showed greater variance between their respective extremity and LM tumors. The power of the paired analysis is diluted in the larger analysis of all extremity tumors versus all LM. These similarities became clearer in the paired analysis (*n* = 4) (Fig. 2B), which demonstrated that the extremity tumors were more likely to cluster with their matching metastasis.

### Pathway analysis identifies upregulation of the AR pathway in metastatic OS

**Fig. 3:**
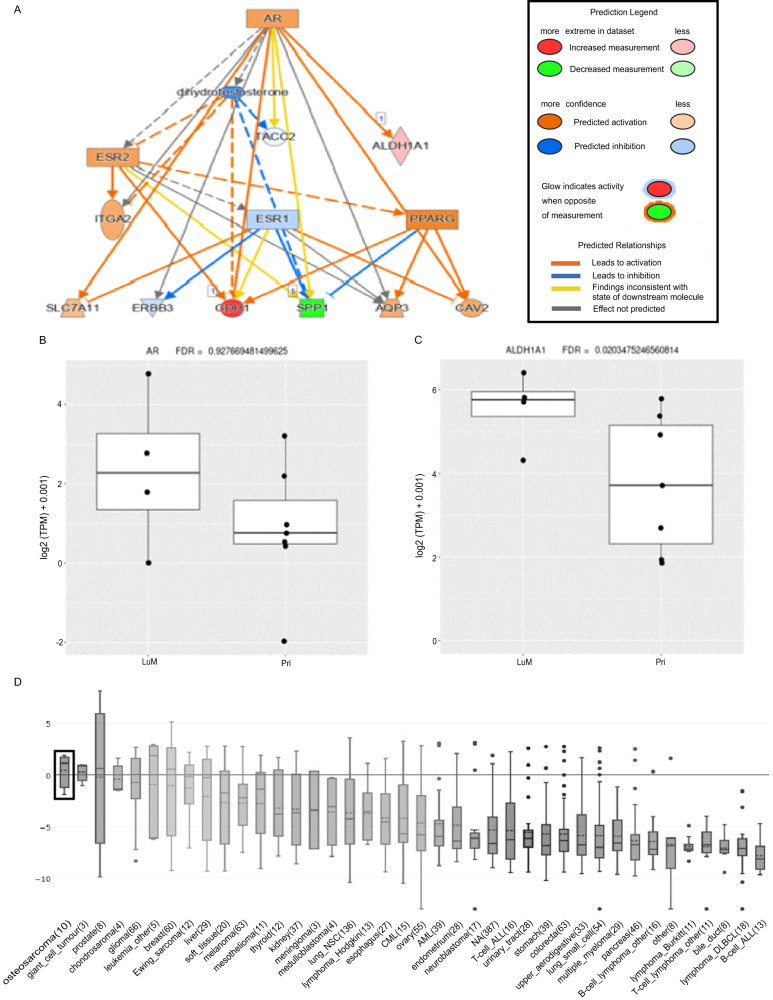
Androgen receptor (AR) is predicted to be an upstream regulator in the lung metastases of OS patients. **A** IPA (Qiagen, v68752261) analysis on all genes (Cut-offs for Exp. Log ratio = −0.58 and 0.58 and FDR = 0.01) revealed genes upregulated in LM compared to extremity tumors were found to be involved in several pathways, including the AR pathway. Several genes downstream of AR (*p*-value = 4.11 × 10^–6^) were found to be significantly upregulated in LM tissue, including aldehyde dehydrogenase (*ALDH*, log2FC = 1.48, FDR = 0.02). **B** *AR* and **C** *ALDH* expression values (log2normCPMs). FDR calculated by Bejamini-Hochberg adjustment is shown. **D** *AR* expression levels across cancer cell subtypes from the Broad Cancer Cell Line Encyclopedia via a waterfall plot based on median expression. OS is outlined in the box.

We performed pathway analysis utilizing Ingenuity Pathway Analysis (IPA) (Qiagen, v68752261) on all genes (cut-offs for Exp. Log ratio = −0.58 and 0.58 and FDR = 0.01) for the paired samples to determine which sets of genes or pathways might be upregulated in the LM compared with their respective extremity tumors. Because all patient-matched extremity tumors clustered similarly with each other in our PCA, all representative extremity samples for a patient were used as a pooled sample in the following comparative analyses. The top molecular/cellular function for both analyses was Cellular Movement (*p*-value = 3.16 × 10^–20^). This was expected given the general mechanisms thought to underlie metastasis from one tissue to another distant site. Interestingly, *AR* was identified as an upstream regulator in the paired LMs compared to the extremity tumors (*p*-value = 4.11 × 10^–6^). This activated upstream regulation for AR was also identified in the unpaired samples (*p*-value = 1.15 × 10^–4^), however this relationship was strongest in the paired samples, demonstrating a unique role for *AR* in the metastatic samples. Interestingly, IPA predicted *ALDH1A1* (log2FC = 1.48, FDR = 0.02) as a downstream target of *AR* (Fig. 3A). Additional analyses for *ALDH1A1* expression levels in the paired dataset reveal significant increases in LM compared to primary and recurrent tumors, however, AR expression differences were not significant (Fig. 3B, C). Despite AR not being significantly upregulated in the LM, there may still be some regulation of ALDH1A1 by AR as has been previously demonstrated in prostate cancer [9]. Using online CCLE data curated by the Broad Institute via a waterfall plot based on median expression, we found that *AR* has been noted to be more highly expressed in OS (Fig. 3D) than in any other malignancies, including prostate cancer. This reinforces *AR* as a promising potential target in OS.

### Aberrant expression of AR and ALDH1A1 is displayed in primary and metastatic OS cells

**Fig. 4:**
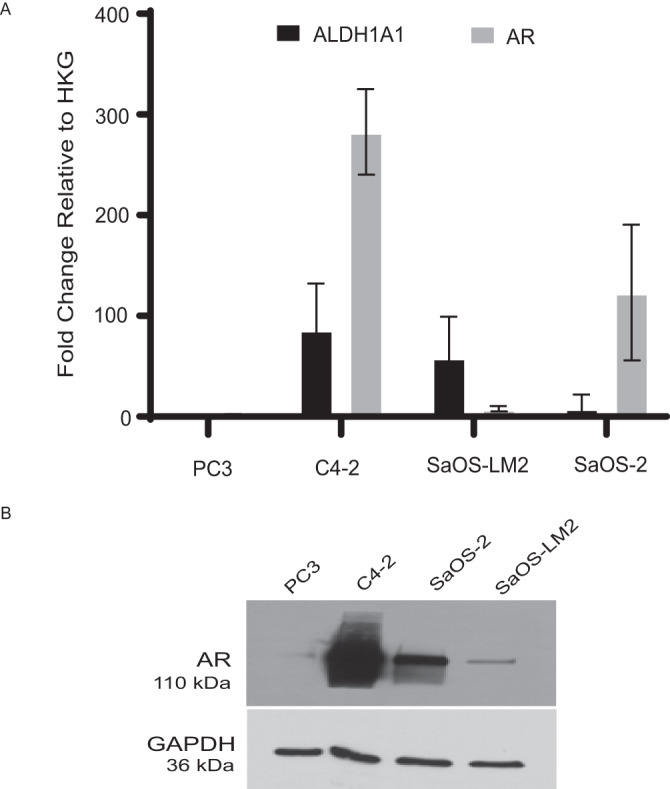
AR expression levels in OS cell lines. **A** The mRNA expression levels of *ALDH1A1* and *AR* were detected by qRT-PCR for the related OS cell lines SaOS-2 and SaOS-LM2. Prostate cancer cell lines PC3 and C4-2 were used as negative and positive controls, respectively. SaOS-2 was found to express high levels of *AR* and relatively low levels of *ALDH1A1*, while SaOS-LM2 was discovered to express low levels of *AR* and high levels of *ALDH1A1*. Data are expressed as mean ± SD (*n* = 3, experiment was performed in triplicate). **B** Western blotting also revealed AR expression at the protein level in both OS cell lines. GAPDH served as loading control, while PC3 and C4-2 served as negative and positive controls, respectively for AR.

Given that AR was predicted to be an upstream regulator in LM from pathway analyses, we utilized an OS cell line that was isolated from a pulmonary metastasis in mice that had been injected with SaOS-2 [30]. Both *ALDH1A1* and *AR* are expressed in both the parental SaOS-2 and isogenic SaOS-LM2 cell lines at the RNA (Fig. 4A) and protein (Fig. 4B) levels. PC3 negative control cells did not express either target. As expected, the highly metastatic SaOS-LM2 and positive control C4-2 cell lines yielded the highest expression levels of *ALDH1A1*. However, unlike the prostate cell line, SaOS-LM2 had relatively low levels of *AR*. The OS cell line with low metastatic potential, SaOS-2, had higher expression of *AR* compared to SaOS-LM2 displaying an inverse relationship between *ALDH1A1* and *AR* in these OS cell lines.

### Inhibition of AR causes a decrease in ALDH1A1 activity in OS cell lines

**Fig. 5:**
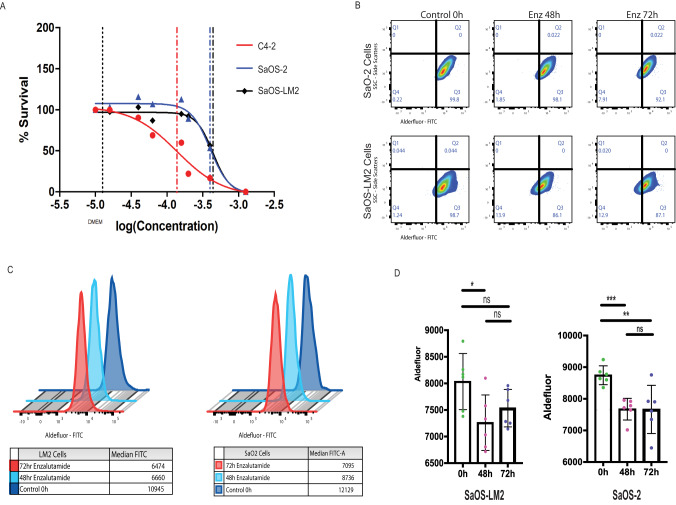
Treatment of human OS cell lines with Enzalutamide displays an inhibitory effect on the activity of ALDH. **A** Enzalutamide was used at fold dilutions starting at 100 µM. Dose-response curve of enzalutamide fold dilutions on C4-2, SaOS-2, and SaOS-LM2. The IC_50_ concentrations of enzalutamide are 98.46 µM 109.7 µM (SaOS-LM2 and SaOS-2 respectively). C4-2 served as a positive control. **B** Human OS cells (SaOS-2 and SaOS-LM2) were cultured in 3 conditions (control: no enzalutamide, 48-h enzalutamide exposure, and 72 enzalutamide exposure), with 6 replicates per condition, and harvested for Aldeflour Assay analysis for ALDH activity using Flow Cytometry. Collected cells in each condition were gated base on ALDH activity. SaOS-2 displayed a constant decrease in ALDH activity at both drugging time points. SaOS-LM2 displayed an initial decrease in ALDH activity after 48 h of drug exposure but showed a slight rebound increase in ALDH activity at the 72-h time point. **C** Median ALDH activity is displayed in overlayed fluorescence graphs for all conditions and cell lines. For both cell lines, longer exposure to enzalutamide presented with a decrease in ALDH activity. **D** ALDH activity is displayed in a bar graph by compiling the median ALDH activity from all respective biological replicates. In SaOS-2, again, there is significant decrease in ALDH activity when comparing 48-h and 72-h drug exposure to untreated control. In SaOS-LM2, there is a significant decrease in ALDH activity in the 48-drug exposure time point, with an observed slight increase in ALDH activity at the 72-h time point. The statistical significance among the different treatment time points in the Aldefluor activity assay was calculated using two-tailed Student’s unpaired *t*-test. Data represent mean ± SD: *p < 0.05; **p < 0.01; ***p < 0.001.

To evaluate the downstream effects of AR inhibition, SaOS-2, and SaOS-LM2 cell lines were treated with enzalutamide for a total of 48 or 72 h. C4-2, an AR-positive castrate-resistant prostate cancer cell line, served as a positive control. Each cell line was treated with its respective IC_50_ enzalutamide concentration which was previously determined through a dosing-curve experiment (Fig. 5A). Enzalutamide inhibited ALDH activity in both experimental and control cell lines at the 48 h time point when compared with the untreated cells (Fig. 5C). Enzalutamide treatment for 48 and 72 h significantly decreased ALDH activity in SaOS-2 compared with untreated cells. There was also a significant decrease in ALDH activity in SaOS-LM2 cells after being treated with enzalutamide for 48 h. However, no cell line had a significant difference in ALDH activity between the 48- and 72-h time points. SaOS-2 demonstrated a continuous decline in the median ALDH activity at longer exposure to enzalutamide (Fig. 5D).

### OS cell migration after enzalutamide treatment

**Fig. 6:**
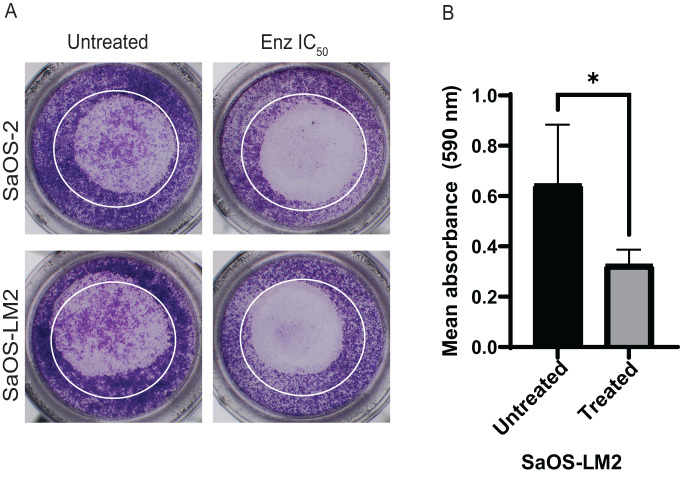
Enzalutamide significantly changes migration of SaOS-LM2 cells after 24-h treatment. OS cell lines were treated with enzalutamide at their respective IC_50_ for 24 h before plating cells in migration assay. All cells were serum-starved for 24 h prior to plating cells on transwell inserts. Transwell plates were incubated for 18 h before staining with crystal violet. **A** Representative images of the transwell inserts were taken using an Olympus SZX16 microscope (5× objective with 2.9× zoom). **B** Bound crystal violet was eluted from each replicate well (*n* = 3) and absorbance was measured. Decreased migration due to treatment was significant for the SaOS-LM2 cell line. Data was analyzed using an unpaired *t*-test with significance defined as *p* < 0.05. Error bars are reported as SEM, each migration assay (*n* = 4) was run separately.

Enzalutamide was used to test the effect of AR inhibition on migration of OS cells with varying metastatic potential. Treatment with enzalutamide for 24 h decreased migration of cells across a transwell membrane in both OS cell lines, regardless of their metastatic potential. This decrease was significant for SaOS-LM2 (Fig. 6) but SaOS-2 cells’ response to enzalutamide was inconsistent and not significant. Inhibition of AR in this in vitro study yielded a statistically significant decrease in migration of metastatic OS cells only.

## Discussion

OS is the most common primary malignancy of bone and predominantly affects patients in the first two decades of life. The most important predictor of survival is the presence or absence of metastatic disease. Like most sarcomas, OS demonstrates a particular tropism for the lung. The presence of LM remains the most important clinical determinant of OS prognosis, and the prognoses of patients with LM have not improved in several decades. The problem of metastatic disease in OS may be addressed with targeted approaches that inhibit the metastatic potential of OS cells.

Here we describe RNA-seq of a series of archival OS tissues and identify several DEGs between primary and recurrent tumors of the extremities and LM tumors. LM tumors demonstrated notable upregulation of many genes compared with the extremity tumor subset. Among the many DEGs we identified, *ALDH1A1* was consistently overexpressed in OS LM. Our previous work has described the importance of ALDH in the metastatic biology of OS [6, 8, 9]. We observed distinct genetic clustering of extremity and LM tumors. Furthermore, in two patient-matched pairs, the primary and recurrent tumors from different patients exhibited more similarities than with their respective LM. These unbiased data support our hypothesis that genetic changes occur during the metastatic process, and that these changes may be consistent among OS LM. These observations suggest that LM may be susceptible to appropriately targeted therapies. To the best of our knowledge, this is the first time that upregulation of the AR pathway has been identified in metastatic OS tissue. Pathway analyses of these samples predicted that AR is an important protein of the metastatic phenotype in OS, including ALDH activity. This finding was validated by measuring ALDH activity and migration of human OS cell lines SaOS-2 and SaOS-LM2 with AR inhibition. ALDH was significantly decreased in both cell types after treatment with enzalutamide for 48 h. Enzalutamide treatment after 24 h also significantly decreased migration of the cell line with higher metastatic potential (SaOS-LM2) in our study. However, this effect was not significant in the primary OS cell line. These findings may be clinically relevant, and several AR inhibitors are used ubiquitously in the treatment of prostate cancer. This case for drug repurposing should be investigated further.

Although our study encompassed a small number of total patients, we observed male predominance (57%) in our sample set, reflective of the higher incidence of OS in peri-pubertal males, in whom androgen levels are very high. Also consistent with current reports, the male patients of this study had a higher incidence of metastases and lower survival than the female population. Previous survival analyses have shown this survival difference between genders is independent of disease stage. It should be noted the average age at diagnosis was higher in both males (31 years) and females (47 years) in this study compared to previous clinical reports [15].

This work provides support for further evaluation into the link between hormonal regulation and OS, especially in the setting of advanced disease. Future studies will validate the genetic targets identified here in tissue microarray using additional frozen patient samples from our biobank. AR can be targeted with other FDA-approved inhibitors and tested in vitro and in vivo. As such, identification of AR pathway upregulation in OS LM tissue samples may provide additional evidence to support AR as a target of novel therapeutics for patients with this disease.

There are limitations to this study. As with most sarcoma studies that employ clinical samples, access to OS clinical samples is limited by the rarity of this disease. Despite this, we are not aware of any studies in the literature with a larger sample size of primary, recurrent, and metastatic OS tumors, including matched sets from the same patients. In order to collect this relatively large sample size, the tissues used in this study were from FFPE samples. Most OS samples are fixed in formalin and decalcified, which undeniably affects the integrity of nucleic acids in these samples. However, we have developed familiarity with these types of samples, as is demonstrated in our prior work [31], and ensured all samples used in this study met stringent quality control standards. Although several chemotherapeutic agents used for treating OS can cause adverse effects, including on the reproductive system [32], we also want to note the potential risks targeting hormonal markers like AR could pose in a pubescent patient. Further mechanistic analyses will need to be elucidated before repurposed enzalutamide will be offered in a clinical setting.

In summary, we demonstrate a potential role of AR and ALDH in the regulation of OS LM. Because OS disproportionately affects male adolescents during puberty, there have been several hypotheses about the role of sex hormones in the development of OS. Our study demonstrates that AR may be directly responsible for the upregulation of chemo-resistant genes, like *ALDH1A1*, ultimately producing the perfect ‘micro-environment’ for OS cells to proliferate, disseminate, and initiate growth as LM. This study provides evidence to support the efficacy of targeting the AR pathway as a novel approach to OS LM treatment.

## Methods

### Patient sample acquisition

Patients with OS treated surgically at our institution between 2002 and 2016 were identified and FFPE archival samples from primary and recurrent sites within an extremity and LM tumors were obtained. Primary tumor samples included biopsies (B) and primary tumor resections (R). Recurrent tumors (C) were local to the primary occurrence. Chart reviews were performed to obtain the pertinent clinical characteristics of each patient. We identified samples from 28 unique patients. Four patients had samples available from both an extremity and LM, 19 patients had samples exclusively from the extremity site, and five patients had samples exclusively from LMs. Among the 38 total samples, there were 14 samples from extremity tumor sites in patients that never developed LM (L), 5 of these extremity tumors were from B and the other 9 were from R. Another 14 samples were from extremity tumor sites in patients that did develop LM (M) with 3 from B, 8 from R, and 3 from C. There was a total of 10 LM samples (LM) included in this study. Sixteen of the 28 patients (57%) were male. Patient histories were obtained up to their most recent follow-up or reported date of death. Of the 12 female patients in this study, only 5 patients ( < 42%) developed metastatic tumors (Table 1). Twelve patients did not receive neoadjuvant chemotherapy (Table 2). Fourteen patients are alive at the time of this publication. Survival for the other 14 patients ranged from two months to seven years from the time of primary diagnosis.

**Table 1:** Patient clinical information and quality assessment.

Sample ID	ID type	Age at diagnosis	Gender	Living status	Sample type	Cellularity (%)
OS-01	LM1	Unknown	Male	Deceased	lung metastasis	50
	LM2				lung metastasis	50
OS-02	LM	5	Male	Alive	lung metastasis	90
OS-04	LM	16	Male	Deceased	lung metastasis	60
OS-05	LM	16	Male	Deceased	lung metastasis	60
OS-06	R(M)	17	Male	Deceased	primary	60
	LM				lung metastasis	90
OS-07	B(M)	14	Male	Deceased	primary biopsy	20
	R(M)				primary	80
	LM				lung metastasis	80
OS-08	R(L)	13	Female	Alive	primary	70
OS-09	B(L)	15	Female	Alive	primary biopsy	60
OS-10	R(L)	15	Female	Alive	primary	40
OS-11	B(L)	38	Female	Alive	primary biopsy	60
OS-12	B(L)	13	Male	Alive	primary biopsy	50
OS-13	R(M)	76	Female	Deceased	primary	90
OS-14	R(M)	84	Female	Deceased	primary	80
OS-15	R(L)	63	Male	Alive	primary	50
OS-16	R(M)	29	Female	Alive	biopsy	80
OS-17	R(L)	60	Female	Deceased	primary	80
	C1(L)				recurrence	80
	C2(L)				recurrence	80
OS-18	B(M)	23	Female	Alive	biopsy	60
OS-19	R(M)	53	Male	Alive	primary	70
OS-20	R(L)	83	Female	Alive	primary	80
OS-21	R(L)	17	Male	Alive	primary	80
OS-22	LM	68	Male	Deceased	lung metastasis	80
OS-23	C(M)	42	Female	Deceased	recurrence	80
	LM				lung metastasis	80
OS-24	R(L)	85	Female	Deceased	primary	80
OS-25	R(L)	31	Male	Alive	primary	90
OS-26	R(L)	15	Male	Alive	biopsy	90
OS-27	R(M)	74	Male	Deceased	primary	90
OS-28	R(M)	13	Male	Deceased	biopsy	90
OS-29	B(M)	58	Male	Deceased	biopsy	90
	C1(M)				biopsy of recurrence	90
	C2(M)				recurrence	70
	LM				lung metastasis	100

**Table 2:** Patient disease progression and treatment history.

Sample ID	# months to recurrence (since primary diagnosis)	# months to LM (since primary diagnosis)	# months to other sites of metastases (since primary diagnosis)	Chemotherapy	Radiation
OS-01	Unknown	Unknown	Unknown	Unknown	Unknown
OS-02	n/a	2	n/a	Neoadjuvant and adjuvant	None
OS-04	n/a	145	36 (contralateral tibia), 180 (GI)	Neoadjuvant and adjuvant	None
OS-05	n/a	17, 20, 58, 62, 63	n/a	Neoadjuvant	None
OS-06	60	26	87 (GI)	Neoadjuvant and adjuvant	None
OS-07	21	9	9 (spine)	Neoadjuvant and adjuvant	Yes, after disease progression
OS-08	n/a	n/a	n/a	Neoadjuvant and adjuvant	None
OS-09	n/a	n/a	n/a	Neoadjuvant and adjuvant	None
OS-10	n/a	n/a	n/a	Neoadjuvant and adjuvant	None
OS-11	n/a	n/a	n/a	None	None
OS-12	n/a	n/a	n/a	Neoadjuvant and adjuvant	None
OS-13	n/a	6	n/a	Adjuvant	None
OS-14	n/a	de novo	de novo (liver)	None	None
OS-15	n/a	n/a	n/a	Neoadjuvant and adjuvant	None
OS-16	n/a	24	n/a	Neoadjuvant and adjuvant	None
OS-17	3	n/a	n/a	None	Prior for breast cancer prior to OS
OS-18	n/a	de novo	n/a	Neoadjuvant and adjuvant	Yes, after disease progression
OS-19	17	22	11 (groin)	None	Yes, after disease progression
OS-20	n/a	n/a	n/a	None	None
OS-21	n/a	n/a	n/a	Neoadjuvant and adjuvant	None
OS-22	7	4	n/a	None	None
OS-23	24	24	n/a	Adjuvant	None
OS-24	n/a	n/a	n/a	None	None
OS-25	n/a	n/a	n/a	Adjuvant	None
OS-26	n/a	n/a	n/a	Neoadjuvant and adjuvant	None
OS-27	n/a	3	n/a	None	Yes, for prostate cancer prior to OS
OS-28	n/a	27	23 (clavicle, elbow), 24 (vertebra, forearm, popliteal fossa)	Neoadjuvant and adjuvant	Yes, after disease progression
OS-29	13, 27	24	48 (arm)	Adjuvant	None

### FFPE sample processing, RNA extraction, and quality control

A board-certified pathologist reviewed hematoxylin and eosin slides from each sample and marked areas with high tumor cellularity and less than 40% necrosis. Subsequently, five to seven 10 µM sections from the paraffin blocks were cut, and the areas of interest were macrodissected with a sterile, disposable No. 15 scalpel (Fisher Scientific, Waltham, MA, USA Cat.# 50822460). RNA extraction was then performed with the AllPrep DNA/RNA FFPE kit (Qiagen, Germantown, MD, USA, Cat.# 80234) according to the manufacturer’s instructions under sterile RNase/DNase-free conditions. RNA concentration was determined with the Qubit 4.0 Fluorometer (Thermo Fisher Scientific, Waltham, MA, USA, Cat.# Q33227) using the Qubit RNA BR Assay Kit (Thermo Fisher Scientific, Q10211). Quality RNA integrity number scores, and fragment sizes (DV200 metrics) were obtained utilizing the Agilent 4200 TapeStation system (Agilent Technologies Inc, Santa Clara, CA, USA, Cat.# G2991BA).

### ecRNA-seq, expression quantification, and normalization

For patient samples which passed quality control tests, we performed ecRNA-seq as previously described with minimal changes [31]. Briefly, sequencing library preparation was performed using a minimum of 40 ng RNA according to Illumina’s TruSeq RNA Access Library Preparation protocol (Illumina, San Diego, CA, USA, Cat.# 20020189). Indexed, pooled libraries were then sequenced on the Illumina NextSeq 500 platform with high-output flow cell-producing stranded, paired-end reads (2 × 75 bp, paired-end). A target count of 40 million reads per sample was used to plan indexing and sequencing runs. For expression quantification and normalization, the RNA transcripts from paired-end FASTQ files were mapped and quantified using kmer–based quasi-mapping with seqBias and gcBias corrections (Salmon v1.1.0, 31-kmer index built from GRCh38 Ensembl v99 transcript annotations) [33]. Transcript-level abundance estimates were collapsed to gene-level estimates using tximport [30]. Gene-level counts or log2normCPM values were implemented for subsequent analyses [34, 35].

### Differentially expressed genes analysis (unsupervised hierarchical clustering)

Differentially expressed genes (DEGs) between patient LM samples and extremity tumor patient samples were calculated using a paired or unpaired DESeq2 v1.24.0 differential gene expression analysis controlling for method of collection (biopsy vs. resection); design ≃ method of collection + condition (extremity/metastasis). This enabled us to test for the effect of extremity tumors in contrast to LM while controlling for the effect of the different collection procedures, which included biopsy or resection. DEGs were defined as genes with an absolute value fold-change ≥ 1.5; FDR ≤ 0.01; and minimum TPM of 1 in at least 10% of the samples. Gene ontology analysis was performed to determine biological processes represented in the upregulated and downregulated gene sets. We performed hierarchical clustering utilizing the heatmap.3 function (https://raw.githubusercontent.com/obigriffith/biostar-tutorials/master/Heatmaps/heatmap.3.R). AR expression across cell lines was examined using Cancer Cell Line Encyclopedia (CCLE) data. Gene expression levels from processed RNA-seq of 1379 cell lines were obtained from CCLE along with the sample information containing primary disease types and subtypes for all samples (version DepMap Public 21Q2). Prior to analysis, all bone cancer cell lines were sorted into their respective subtypes: Osteosarcoma, Chondrosarcoma, and Ewing Sarcoma. Median AR expression values and quartile ranges for primary diseases and bone subtypes were calculated in base R and plotted using ggplot2 v.3.3.5. Pathway analyses were performed using IPA (Qiagen, v68752261). Cut-offs for Exp. Log ratio = −0.58 and 0.58 and FDR = 0.01.

### Cell lines and culture conditions

Human primary OS SaOS-2 (ATCC, Manassas, VA, USA, Cat.# HTB-85) and dedifferentiated chondrosarcoma HT-1080 (ATCC, Cat.# CCL-121) cell lines were purchased from American Type Culture Collection. SaOS-2 was developed from the OS primary tumor of an 11-year-old female. While HT-1080 was characterized as a fibrosarcoma of bone from a 35-year-old male, the cell line has since been reported to possess an *IDH1* mutation which suggests that a diagnosis of dedifferentiated chondrosarcoma is more appropriate [36]. The metastatic SaOS-LM2 OS cell line was gifted from Dr. Eugenie Kleinerman (MD Anderson Cancer Center, Houston, TX, USA). SaOS-LM2 is a LM subline developed by repeated intravenous ‘cycling’ of SaOS-2 in a murine model. SaOS-LM2 demonstrated a phenotype with a higher metastatic potential than SaOS-2 [37]. Cell lines were authenticated at the MD Anderson Cancer Center Cytogenetics and Cell Authentication Core. The AR-positive castrate-resistant, C4-2, and AR-negative, PC3, prostate cell lines were generously provided by Dr. Zhou Wang (UPMC Department of Urology, Pittsburgh, PA, USA). Cells were determined to be mycoplasma-free after testing with MycoAlert Plus mycoplasma kit (Fisher Scientific, Cat.# NC0529908). SaOS-2 and SaOS-LM2 OS cell lines were cultured in complete media (Dulbeco’s Modified Eagle Medium (Gibco, Billings, MT, USA, Cat.# 11995065) + 10% fetal bovine serum (FBS) + 1% MEM Non-Essential Amino Acids Solution (Gibco, Cat.# 11140050) + 1% MEM Vitamin Solution (Gibco, Cat.# 11120052) + 1% Penicillin-Streptomycin Solution (Corning, Corning, NY, USA, Cat.# MT30001CI)). HT-1080 cells were cultured in Dulbeco’s Modified Eagle Medium supplemented with 10% FBS and 1% penicillin-streptomycin. C4-2 and PC3 were cultured in RPMI-1640 medium (Gibco, Cat.# A1049101) supplemented with 10% FBS and 1% penicillin/streptomycin. All cells were maintained in a humidified atmosphere.

### Detecting AR with western blotting OS cell lines

Protein lysates of PC3, C4-2, SaOS-2, and SaOS-LM2 cell lines were extracted in triplicate with RIPA Lysis Buffer (Sigma-Aldrich, St. Louis, MO, USA, Cat.# R0278) plus Halt Protease and Phosphatase Inhibitor (Thermo Fisher Scientific, Cat.# 78442). Protein concentrations were measured using the Pierce BCA Protein Assay Kit (Thermo Fisher Scientific, Cat.# 23225). Equal amounts of protein (20 µg) were loaded and run on a 4–20% SDS polyacrylamide gel (Bio-Rad, Hercules, CA, USA, Cat.# 4561094). Electrophoresis was performed at 115 V for 70 min. Mini Trans-Blot Cell System (Bio-Rad, Cat.# 1703930) was used to transfer protein (100 V for 60 min) from gel to nitrocellulose membrane (Bio-Rad, Cat.# 1620112). The membrane was blocked with 5% milk at room temperature for 1 h, then incubated with primary antibodies at 4 °C overnight. Antibodies for AR (1:1000 dilution) and GAPDH (1:5000 dilution) were purchased from Abcam (Cambridge, UK, Cat.# ab133273) and Cell Signaling Technology (Danvers, MA, USA, Cat.# 5174 S), respectively. Next, the membranes were incubated with secondary antibody (1:3000) (Bio-Rad, Cat.# 1706515) in 2.5% milk for 1 h at room temperature. Blots were washed 3 times for 6 min each after primary and secondary antibody incubations. Western blots were incubated with ECL (Thermo Fisher Scientific, Cat.# 32106) and imaged for chemiluminescence with the Kodak X-Omat 2000 Processor on BioBlot BXR film (Laboratory Product Sales Inc., BX57). Detection of AR with a western blot was replicated three separate times.

### Cell line RNA extraction and quantitative real-time polymerase chain reaction (qRT-PCR)

Biological triplicates of RNA were collected from SaOS-2, SaOS-LM2, PC3, and C4-2 cell lines (1.25 × 10^6^ cells) according to the manufacturer protocol (Qiagen RNeasy Mini Kit, 74106). The highly metastatic PC3 and C4-2 prostate cell lines were used as negative and positive AR controls, respectively, based on their androgen-sensitivity. In this study, 1 µg of total RNA per sample was used to synthesize the first strand cDNA using iScript reagent (Bio-Rad, Cat.# 1708890) in a total volume of 20 µL. Amplification of triplicate cDNA template samples for the target genes were performed with denaturation for 15 min at 95 °C, followed by 45 cycles of denaturation at 94 °C for 15 s, annealing at 55 °C for 30 s, and extension at 72 °C for 30 s using the CFX Opus 384 real-time PCR instrument (Bio-Rad, Cat.# 12011452). Primers were designed for *AR* (F: AATCCCACATCCTGCTCAAG, R: AAGTCCACGCTCACCATG) and (F: AGCAGGAGTGTTTACCAAAGA, R: CCCAGTTCTCTTCCATTTCCAG). Cycle threshold (Ct) values were normalized to *ANKRD28* (F: TTGGAGTGCCTAAACCTTCTG, R: AGGTCATTCACACTTGCTCC), *GAPDH* (F: ACATCGCTCAGACACCATG, R: TGTAGTTGAGGTCAATGAAGGG), and *SYMPK* (F: CTTCACCAAGGTTGTGCTGGAG, R: GCGCTTGAAGATCAGGTCTCGA). The changes in fluorescence of SYBR green dye in every cycle were monitored and calculated by the Bio-Rad CFX384 system software and the Ct for each reaction. The relative amount of PCR products generated from each primer set was determined based on the threshold cycle or Ct value. PCR analysis was performed on each cDNA in triplicate. The method was also performed in triplicate. All primers were supplied by Integrated DNA Technologies (Coralville, IA, USA).

### Testing the effects of enzalutamide on cell viability

C4-2, SaOS-2, and SaOS-LM2 cells lines were harvested after reaching 80% confluence, and 10^5^ cells were plated in 6-well plates for 24 h. C4-2 was used as a positive control for the expression of AR. Cells were then treated with enzalutamide (Selleck Chemical LLC, Houston, TX, USA, Cat.# S1250) at fold dilutions from 100 to 3 µM. In addition to enzalutamide treatment, untreated and methanol-treated (positive control) conditions were also tested. Each condition was performed in triplicate. Cells were incubated in their respective conditions for 72 h, after which they were harvested and counted for viability with Trypan blue (Bio-Rad, Cat.# 1450022).

### Determining ALDH activity in OS cell lines using fluorescence-activated cell sorting (FACS) analysis

SaOS-2 and SaOS-LM2 cell lines (10^6^) were cultured as described above and plated in 60 mm dishes (Fisher Scientific, Cat.# 08772B) for 24 h before any drug intervention to ensure adhesion of cells. Plated cells were then treated with enzalutamide, at IC_50_ concentrations (SaOS-2: 109.7 µM and SaOS-LM2: 98.46 µM) for 48 and 72 h. Each group included three technical triplicates for analyses. At the end of the treatment periods, cells were harvested with Tryple Express (Fisher Scientific, Cat.# 12604021). The ALDEFLUOR™ kit (Stemcell Technologies, Cambridge, MA, USA, Cat.# 01700) was used to analyze the cells with ALDH enzymatic activity according to the manufacturer’s protocol as described previously [38]. Briefly, cells were incubated in the ALDEFLUOR™ assay buffer containing the ALDH substrate BAAA at 37 °C for 45 min. 7- Aminoactinomycin D (7-AAD) dye (Thermo Fisher Scientific, Cat.# A1310) was added to stain dead cells. Stained cells were analyzed using BD LSR Fortessa (BD Biosciences, San Jose, CA, USA) with Flowjo software (Version 10.5.2+, Flowjo LLC). Three separate ALDEFLUOR™ assays were performed.

### Testing the effects of enzalutamide on cell migration

The two OS cell lines with varying metastatic potentials, SaOS-2 and SaOS-LM2, and HT-1080, a highly metastatic chondrosarcoma cell line, were plated in 60 mm dishes at 10^6^ cells and serum-starved for 24 h prior to their harvest for cell migration assays. Treated cells had their respective IC_50_ concentrations of enzalutamide (Selleck Chemical LLC, Cat.# S1250) added to the serum-free media during this 24-h incubation. After 24 h of serum-starvation, with or without enzalutamide treatment, cells were plated in triplicate on 6.5 mm transwell permeable supports at a density of 5 × 10^4^ cells, in 24-well plates (Corning, Cat.# 07200174). The supports (upper chamber) held a final total volume of 150 µl serum-free, cell suspension. The (lower chamber) wells of the 24-well plate held 800 µl of the cell’s respective media either with or without FBS. Wells with serum-free media in the lower chambers served as discrete negative controls for each group of the study. Conditions were run in triplicate. Cells were incubated at 37 °C (5% CO_2_) for 18 h, at which point the inserts were stained with crystal violet to terminate the migration assay. This migration assay was repeated four times.

### Quantifying migrated cells with crystal violet staining

Following the migration assay of SaOS-2, SaOS-LM2, and HT-1080, transwell supports were washed twice with Dulbeco’s phosphate-buffered saline (Gibco, Cat.# 14190144) and cells that did not migrate were removed from the upper chamber using a moistened cotton swab. Migrated cells that adhered to the lower surface of the support were stained with crystal violet (Fisher Scientific, Cat.# C58125) for 10 min. Transwell supports were washed with Dulbeco’s phosphate-buffered saline three times and air-dried. Once dry, bright field images were taken using Olympus cellSens Dimension software (Version 2.1) on an Olympus SZX16-ILLT microscope (Olympus, Tokyo, Japan) with 5× objective lens and 2.9× zoom. The crystal violet bound to migrated cells was eluted from the supports by pipetting 400 µl of 33% acetic acid (Fisher Scientific, Cat.# A38500) into each upper chamber and shaking the plates for 10 min. Half of the eluent for each sample, 200 µl, was transferred to a 96-well clear microplate (Corning, Cat.# 3595) and the absorbance at 590 nm was determined using the Tecan Infinite M200 plate reader (Tecan Group Ltd., Männedorf, Switzerland) and Tecan i-control software (Version 3.91.0). Standard curves were generated for each cell line used in the migration assay and were used to calculate the cell concentration from the experimental absorbance measurements.

### Statistics

A two-tailed Student’s unpaired *t*-test was used to find the statistical significance among the different treatment time points in the Aldefluor activity assay as well as between respective treated and untreated cells in the migration assay (Version 9.2.0, GraphPad Prism). Unless otherwise noted, data represent mean ± SD: *p < 0.05; **p < 0.01; ***p < 0.001.

## Data Availability

RNA-sequencing data have been deposited in NCBI’s Gene Expression Omnibus and are accessible through GEO Series accession number GSE220538.
